# Hand and wrist injuries among collegiate athletes vary with athlete division

**DOI:** 10.1186/s40621-021-00363-5

**Published:** 2021-12-14

**Authors:** Kathleen A. Holoyda, Daniel P. Donato, David A. Magno-Padron, Andrew M. Simpson, Jayant P. Agarwal

**Affiliations:** Division of Plastic and Reconstructive Surgery, University of Utah, School of Medicine, 30 N 1900 E, 3B400, Salt Lake City, UT 84132 USA

**Keywords:** Injury rates, Hand injuries, Wrist injuries, NCAA, College, Division I, Division II, Division III

## Abstract

**Background:**

The rates, severity and consequences of hand and wrist injuries sustained by National Collegiate Athletic Association athletes are not well characterized. This study describes the epidemiology of hand and wrist injuries among collegiate athletes competing in different divisions.

**Methods:**

The National Collegiate Athletic Association Injury Surveillance Program (NCAA-ISP) was accessed from 2004 to 2015 for the following sports: baseball, basketball, football, ice hockey, lacrosse, soccer, wrestling, field hockey, gymnastics, softball and volleyball. The data were used to identify all hand and wrist injuries, the specific injury diagnosis, mean time loss of activity following injury, and need for surgery following injury. These were then stratified by gender. Descriptive statistics were performed to examine the association between sports, event type and division. Student's t test was used to calculate p-values for independent variables. Chi-Square test was used to calculate odds ratio. *P* < 0.05 was considered significant.

**Results:**

103,098 hand and wrist injuries were reported in in the studied NCAA sports from 2004 to 2015. Male athletes sustained 72,423 injuries (6.01/10,000 athlete exposure) and female athletes sustained 30,675 injuries (4.13/10,000 athlete exposure). Division I athletes sustained significantly more injuries compared to divisions II and III. Overall, 3.78% of hand and wrist injuries required surgical intervention. A significantly higher percentage of division I athletes (both male and female) underwent surgical intervention compared to divisions II and III. The mean time lost due to hand and wrist injury was 7.14 days for all athletes. Division I athletes missed the fewest days due to injury at 6.29 days though this was not significant.

**Conclusions:**

Hand and wrist injuries are common among collegiate athletes. Division I athletes sustain higher rates of injuries and higher surgical intervention rates, while tending to miss fewer days due to injury. Improved characterization of divisional differences in hand and wrist injuries can assist injury management and prevention.

## Introduction

More than 460,000 National Collegiate Athletic Association (NCAA) student athletes participate in 24 different sports every year (Irick 2016). Approximately 25% of all sport-related injuries involve the hand or wrist (Amadio 1990; Avery et al. 2016; Simpson and McQueen 2006). Despite the high incidence of these injuries, little is published regarding hand and wrist injuries sustained during collegiate athletics and how the injuries are managed.

As a part of its mandate to ensure the health and safety of student athletes, the NCAA created the Injury Surveillance System in 1982 to collect data on injuries sustained during its sporting events (NCAA 2018). Since 2004, the Datalys Center for Sports Research and Prevention, Inc (Indianapolis, IN) began collecting and maintaining the Injury Surveillance Program (ISP) based on reports filed by NCAA athletic trainers. Since its inception, the data collected by the ISP has been used to investigate injury patterns across varying collegiate sports. Despite the number of publications analyzing the ISP, surprisingly little has been published on hand and wrist injuries sustained during collegiate athletics and how the injuries are managed.

We recently published our analysis of hand and wrist injuries in collegiate athletes using the ISP, with specific focus on sport-specific rates of injury while stratifying by event type and gender. We demonstrated the highest hand and wrist injury rates were in men’s and women’s ice hockey, that men were more likely to sustain such injuries and more likely to miss time from their sport than their female counterparts (Simpson et al. 2020). Our results supported the existing ISP-based literature, which also found similar discrepancies in injury rates between gender across different sports (Dalton et al. 2015; Fraser et al. 2017; Kerr et al. 2011; Zuckerman et al. 2015). While our publication provided new insight into hand and wrist injuries in collegiate athletes, it was limited by not investigating how the different NCAA divisions may have impacted our findings. Analyzing divisional differences that may exist in varying injuries, such as hand and wrist injuries, can provide insight into why these differences may exist and how the management of such injuries can be adjusted to achieve better, safer outcomes in collegiate athletes.

The NCAA divisions were created in 1973 to align like-minded campuses in the areas of philosophy, competition and opportunity (NCAA 2021a). Division I athletics are considered to represent the highest level of collegiate competition. Coaches, teammates and internal factors pressure division I athletes to continuously perform at an elite level and can influence their desire for early return to play. Additionally, division I athletes are more likely to play professionally and thus their physical characteristics better represent those seen in professional sports; stronger and faster players can generate greater forces during contact and result in higher rates of injury (Wilcox et al. 2014). These differences between division I athletes and those in other divisions led us to hypothesize that division I athletes would experience higher rates of injury and surgical intervention while missing the fewest days from participating in their sport. As such, the purpose of this study was to further examine the epidemiology of hand and wrist injuries sustained by collegiate student athletes within the NCAA divisions based on information provided by the NCAA-ISP. We specifically sought to compare injury rates, the time lost as a result of injury and the rate of surgical intervention between divisions.

## Methods

This study was deemed exempt from review by the Institutional Review Board on the basis of anonymous, de-identified information. The study protocol was reviewed and approved by the Datalys Center for Sports Injury and Prevention, Inc. (Indianapolis, IN) and by the NCAA.

### Database

We queried the National Collegiate Athletic Association Injury Surveillance Program (NCAA-ISP) from 2004 to 2015. The database is composed of two different data sets, one from 2004–2009 and the second from 2010–2015. Both data sets contain slightly different variables, collection methods and participating teams (Kerr et al. 2014). The data set began in 2004 with the ISP collecting data on 15 core sports and adding additional sports over time. The ISP depends on a convenience sample composed of health information from male and female athletes from divisions I, II, and III that is voluntarily submitted by team athletic trainers from NCAA-sponsored teams. Club collegiate teams were not included in the database. In the event of an injury, athletic trainers provide a thorough report on the specific injury sustained and its circumstances such as the mechanism of injury and the type of event (practice or competition). The injuries reported to the NCAA-ISP were sustained during an organized intercollegiate practice or competition and was identified by an athletic trainer or physician. Athlete exposure (AE) refers to an individual athlete’s participation in school-sanctioned practice or competition. Any injury sustained during activities outside an organized practice or event were not captured in the database (Kerr et al. 2014). Time loss injuries are defined as any injury that results in the restriction of an athlete from participating in practice or competition for at least one day. Non-time-loss injuries are defined as any injury that did not result in restriction of play beyond the day of injury. The recording of non-time-loss injuries is a notable difference between the 2004–2009 and 2010–2015 eras such that the recording of non-time-loss injuries began in 2010. This change in injury reporting was uniform across all divisions and genders, so this change should not affect the findings of the study. The two eras have been combined in similar analyses previously (Simpson et al. 2020; Kerr et al. 2014; Chorney et al. 2017).

### Study design

We performed a retrospective, epidemiological cross-sectional analysis of hand and wrist injuries using information from the 2004–2015 database. We separated injuries by male and female sports, as well as by NCAA division. Men’s sports included baseball, basketball, football, ice hockey, lacrosse, soccer, and wrestling. Women’s sports included basketball, field hockey, gymnastics, ice hockey, lacrosse, soccer, softball, and volleyball. All hand and wrist injuries identified were included. We did not include fractures of the distal radius and ulnar styloid in our analysis as the ISP does not accurately differentiate between proximal or distal location of forearm bone fractures. The ISP assigns all injury events a unique identifier that include the year of injury, sport, and collegiate division. Within an injury event, an athlete may experience multiple injuries however they will remain under the same injury event, in turn, it is not possible for the authors to determine if there are multiple injuries included in the same event.

In order to simplify data analysis, we categorized injuries into fractures and dislocations, and soft tissue injuries. The five categories for fractures and dislocations include: Dislocation-phalanx, phalangeal fracture, metacarpal fracture, scaphoid fracture and other carpal fracture. The 11 soft tissue categories include the following: Ligamentous injury—phalanx, ligamentous injury—triangular fibrocartilaginous complex (TFCC) /Distal radioulnar joint (DRUJ) /Scapholunate, ligamentous injury—other/wrist sprain, contusion—hand/finger, contusion—wrist, Tendon—phalanx, Tendon—wrist, wrist tenosynovitis, infection, nailbed, soft tissue injury—other.

### Statistical analysis

The data presented in the manuscript is representative of weighted, national estimates. The ISP provides the data with associated weights for each event in each sport and division. These weights are updated each academic year and are based on the sport and division so that year-to-year variation in the sampled teams accurately reflects the total population of student athletes. This weighting allows the data to be interpreted as national estimates based on the convenience sample of schools, sports and events, as reported by the athletic trainers. The sample weight calculation was based on the following formula: Weight_*ijk*_ = (# ISS Schools_*ijk*_/# Sponsoring schools_*ijk*_)^−1^ where Weight_*ijk*_ is the weight for sport *i* in division *j* in year *k*. The ISP has been validated previously, revealing that it captures over 88% of all time loss injuries that required medical attention (Kerr et al. 2014). Based on this, post-stratification weights were adjusted by a factor of 0.883^–1^ to increase the accuracy of injury reporting in the ISP (Kerr et al. 2014).

The rates provided in the manuscript are reported per 10,000 athlete exposure. All exposures are captured in this dataset, allowing us to calculate odds ratio and 95% Wald confidence intervals using the Chi-Square test. 95% confidence intervals were created from the weighted frequencies. All confidence intervals not containing 1 were considered statistically significant. We used the student *t* test to determine *p* values for independent variables; values less than 0.05 were used to determine significance. We performed all statistical analyses using SAS Version 9.4 (Cary, North Carolina).

## Results

### Rates of injuries

There were 4422 hand and wrist injuries recorded by the ISP from 2004 to 2015. After appropriate weighting was applied, there were 103,098 hand and wrist injuries in male and female sports across all NCAA divisions from 2004 to 2015. This corresponds to a rate of 5.33/10,000 AE (95% CI 5.17, 5.48) in all athletes in all divisions. The highest injury rate occurred in division I athletes with a rate of 6.14/10,000 AE (CI 5.79, 6.48), followed by division III athletes (5.25/10,000 AE (CI 4.93, 5.57)) and division II athletes (4.17/10,000 AE (CI 3.75, 4.59)). This trend was preserved after stratifying by gender such that male (7.05/10,000 AE (CI 6.62, 7.49)) and female (4.47/10,000 AE (CI 3.90, 5.04)) division I athletes had the highest rates of hand and wrist injury while male (5.82/10,000 AE (CI 5.39, 6.25)) and female (3.38/10,000 AE (CI 2.73, 4.04)) division II athletes had the lowest rates of hand and wrist injury. We summarized this data in Table 1.

**Table 1:** Total hand and wrist injury rate by division and gender

Division	Injury rate/10,000 athlete exposures^a^	95% CI		OR (between divisions)	95% CI		*P* value
All sports							
Division I	6.14	5.79	6.48	Ref	Ref	Ref	Ref
Division II	4.17	3.75	4.59	0.62	0.61	0.63	< 0.01
Division III	5.25	4.93	5.57	0.79	0.78	0.80	< 0.01
All divisions	5.33	5.17	5.48	–	–	–	–
Men's sport							
Division I	7.05	6.62	7.49	Ref	Ref	Ref	Ref
Division II	4.62	4.08	5.17	0.65	0.64	0.67	< 0.01
Division III	5.82	5.39	6.25	0.83	0.81	0.84	< 0.01
All Divisions	6.01	5.80	6.21	–	–	–	–
Womens sports							
Division I	4.47	3.90	5.04	Ref	Ref	Ref	Ref
Division II	3.38	2.73	4.04	0.76	0.73	0.78	< 0.01
Division III	4.26	3.78	4.75	0.95	0.92	0.98	0.01
All Divisions	4.13	3.88	4.37	–	–	–	–

^a^Athlete exposure include practice and competition

### Types of injuries

The types of hand and wrist injuries sustained by collegiate athletes varied widely. The most common type of injury overall were ligamentous injuries of the phalanx and had an overall rate of 1.42/10,000 AE (CI 1.32, 1.51). Ligamentous injuries of the phalanx were also the most common injuries seen within each division: Division I (1.72/10,000 AE (CI 1.56, 1.88)), division II (0.93/10,000 AE (CI 0.76, 1.1)), division III (1.41/10,000 AE (CI 1.26, 1.57)). The most common fractures sustained among all NCAA divisions were metacarpal fractures with a rate of 0.51/10,000 AE (CI 0.45, 0.56); this was also evident in division I (0.54/10,000 AE (CI 0.45, 0.63)) and division II (0.51/10,000 AE (CI 0.38, 0.64)) athletes. Phalangeal fractures were the most common fracture in division III athletes (0.51/10,000 AE (CI 0.40, 0.61)). We demonstrate the specific injuries for each division in Table 2.

**Table 2:** Rates of hand and wrist injuries by diagnosis

	Overall			Division I			Division II			Division III		
	Injury rate/10,000 athlete exposures^a^	95% CI		Injury rate/10,000 athlete exposures^a^	95% CI		Injury rate/10,000 athlete exposures^a^	95% CI		Injury rate/10,000 athlete exposures^a^	95% CI	
Dislocation—phalanx	0.42	0.37	0.48	0.46	0.39	0.54	0.45	0.31	0.59	0.36	0.28	0.44
Phalangeal fracture	0.47	0.41	0.53	0.54	0.44	0.63	0.32	0.22	0.42	0.51	0.40	0.61
Metacarpal fracture	0.51	0.45	0.56	0.54	0.45	0.63	0.51	0.38	0.64	0.47	0.40	0.54
Scaphoid fracture	0.11	0.08	0.14	0.10	0.06	0.14	0.09	0.03	0.15	0.14	0.09	0.18
Other carpal fracture	0.08	0.05	0.10	0.09	0.05	0.14	0.07	0.03	0.11	0.06	0.03	0.09
Ligamentous injury—other wrist sprain	0.69	0.62	0.76	0.80	0.69	0.91	0.39	0.27	0.50	0.77	0.64	0.90
Ligamentous injury—phalanx	1.42	1.32	1.51	1.72	1.56	1.88	0.93	0.76	1.10	1.41	1.26	1.57
Ligamentous injury—TFCC, DRUJ, Scapholunate	0.10	0.07	0.13	0.12	0.07	0.16	0.08	0.02	0.14	0.09	0.04	0.14
Tendon—phalanx	0.19	0.15	0.23	0.16	0.11	0.20	0.23	0.12	0.35	0.19	0.13	0.25
Tendon—wrist	0.11	0.08	0.14	0.12	0.08	0.16	0.12	0.06	0.18	0.09	0.05	0.14
Contusion—hand/finger	0.77	0.69	0.85	0.90	0.77	1.03	0.60	0.41	0.78	0.75	0.62	0.88
Contusion—wrist	0.16	0.12	0.19	0.21	0.14	0.27	0.09	0.03	0.15	0.14	0.10	0.20
Nailbed	0.06	0.03	0.09	0.09	0.04	0.15	0.03	-0.02	0.08	0.05	0.01	0.08
Wrist tenosynovitis	0.08	0.05	0.11	0.06	0.03	0.08	0.11	0.01	0.21	0.08	0.04	0.12
Soft tissue injury	0.16	0.13	0.20	0.21	0.15	0.28	0.15	0.08	0.22	0.13	0.08	0.18
Infection	0.01	0.00	0.02	0.02	0.00	0.03	0.00	0.00	0.00	0.01	0.00	0.02

^a^Athlete exposure include practice and competition

### Management of hand and wrist injuries in collegiate athletes

The majority of hand and wrist injuries were managed non-surgically. Of all NCAA student athletes that sustained hand and wrist injuries between 2004 and 2015, 3.78% underwent surgical treatment. A significantly higher percentage of division I student athletes (4.45%) underwent surgical intervention for their hand and wrist injuries compared to division II (3.56%) and division III (3.12%) student athletes (*p* < 0.01). This pattern was consistent across male and female sports. Division I male student athletes with hand and wrist injuries underwent surgical intervention at a rate of 4.88%, while 4.37% of division II male student athletes underwent surgery and 3.36% of division III male student athletes underwent surgical intervention (*p* < 0.01). For division I female student athletes that sustained hand and wrist injuries, 3.22% underwent surgical intervention, while 1.65% division II female student athletes and 2.57% division III female student athletes underwent operative treatment for their hand and wrist injuries (*p* < 0.01). We summarize this data in Table 3.

**Table 3:** Hand and wrist injuries requiring surgery

Division	Injuries requiring surgery (%)	Surgery rate/10,000 athlete exposure^a^	95% CI		OR (between divisions)	95% CI		*P* value
All sports								
Division I	4.45	0.27	0.25	0.30	Ref			
Division II	3.56	0.15	0.10	0.20	0.54	0.50	0.60	< 0.01
Division III	3.12	0.16	0.13	0.20	0.60	0.56	0.65	< 0.01
All divisions	3.78	0.20	0.19	0.21	–			
Men's sport								
Division I	4.88	0.34	0.31	0.38	Ref			
Division II	4.37	0.20	0.13	0.28	0.59	0.53	0.65	< 0.01
Division III	3.36	0.2	0.15	0.24	0.57	0.52	0.62	< 0.01
All divisions	4.22	0.25	0.24	0.27	–			
Women’s sports								
Division I	3.22	0.14	0.11	0.18	Ref			
Division II	1.65	0.06	0.00	0.11	0.39	0.31	0.50	< 0.01
Division III	2.57	0.11	0.06	0.16	0.76	0.65	0.89	0.01
All divisions	2.65	0.11	0.09	0.13	–			

^a^Athlete exposure include practice and competition

### Mean time loss following hand and wrist injuries

Most hand and wrist injuries sustained by NCAA athletes result in less than one day lost of athlete participation. The mean time lost among all collegiate athletes was 7.14 days (CI 6.02, 8.25), with division I student athletes missing 6.29 days (CI 5.39, 7.19), division II student athletes missing 7.96 days (CI 6.38, 9.54) and division III student athletes missing 7.86 days (CI 5.09, 10.62). Male division I student athletes missed the fewest number of days on average with a mean of 5.66 days (CI 4.79, 6.52), male division II student athletes missed a mean of 8.37 days (CI 6.35, 10.39) and male division III student athletes missed a mean of 8.99 days (CI 4.99, 12.99) from their hand and wrist injuries. The trend was not consistent among female collegiate student athletes. Division I female student athletes missed a mean of 7.87 days (CI 5.57, 10.18), while division II female student athletes missed a mean of 7.04 days (CI 4.62, 9.47) and division III student athletes missed a mean of 5.39 days (CI 4.32, 6.46). We summarized this data in Table 4.

**Table 4:** Mean time loss following total hand and wrist injuries

Sport	< 1 day	1–7 days	8–14 days	15–30 days	30–60 days	> 60 days	Mean time lost (days)	95% CI (days)	
*Time lost to injury (%)*									
All sports									
Division I	57.2	25.0	7.2	4.0	4.9	1.7	6.29	5.39	7.19
Division II	44.3	32.2	7.7	7.2	7.5	1.1	7.96	6.38	9.54
Division III	45.1	34.4	10.5	5.3	3.3	1.5	7.86	5.09	10.62
All Divisions	50.5	29.7	8.5	5.0	4.8	1.5	7.14	6.02	8.25
Male sports									
Division I	59.2	23.7	6.8	3.6	5.2	1.5	5.66	4.79	6.52
Division II	45.1	30.8	8.4	6.3	8.0	1.4	8.37	6.35	10.39
Division III	46.1	33.2	9.8	5.9	3.4	1.7	8.99	4.99	12.99
All divisions	52.0	28.4	8.2	4.9	5.0	1.6	7.29	5.78	8.80
Female sports									
Division I	52.2	28.2	8.0	5.1	4.2	2.3	7.87	5.57	10.18
Division II	42.4	35.3	6.1	9.4	6.4	0.4	7.04	4.62	9.47
Division III	49.1	33.1	10.8	3.4	2.7	0.9	5.39	4.32	6.46
All divisions	46.9	32.8	9.2	5.4	4.1	1.5	6.79	5.61	7.97

## Discussion

The NCAA has been monitoring injuries of collegiate athletes for almost four decades and its most recent iteration, the Injury Surveillance Program, has been used to analyze patterns of injuries in 25 collegiate sports (Dalton et al. 2015; Zuckerman et al. 2015; Dick et al. 2007a, b, c; Agel et al. 2007; Kerr et al. 2017, 2018a, b; Bartels et al. 2019; Clifton et al. 2018; Pierpoint et al. 2019; Lynall et al. 2018). Considering that hand and wrist injuries account for approximately 25% of all sport-related injuries (Amadio 1990; Avery et al. 2016; Howse 1994), it was unexpected that hand and wrist injuries in collegiate athletes using the ISP has been poorly studied (Bartels et al. 2019; Bowers et al. 2008; Deckey et al. 2020). Our recent publication studied the epidemiology of such injuries across major collegiate sports stratified by gender and competition. We revealed that male athletes experience injuries with more frequency and severity than female athletes and injuries were more likely to occur in competition than practice (Simpson et al. 2020). Our results have been supported by other publications evaluating the epidemiology of varying injuries using the ISP (Dalton et al. 2015; Fraser et al. 2017; Kerr et al. 2011; Zuckerman et al. 2015). A limitation in our prior analysis was failing to investigate how hand and wrist injuries may vary across the three NCAA divisions.

It is generally accepted that division I athletics represent the most skilled athletes, teams and highest level of play; to our knowledge, there are no studies that establish this perceived difference yet. The NCAA differentiates its divisions through varying requirements for schools to remain competing in each division; division I has the strictest requirements (NCAA 2021a). Monetary differences between divisions may indirectly support the presumed differences. Of the $1 billion in revenue generated by the NCAA in 2017, division I programs received 60% of that revenue while division II and III programs accounted for 4.37% and 3.18%, respectively (NCAA 2021b). Of the $4.2 billion available for athletic scholarships, division I and II schools receive 65% and 18%, respectively, while athletic scholarships are prohibited in division III programs (O’Rourke 2020). Lastly, the NCAA’s recent analysis on the draft outcomes in professional sports also supports the superiority of division I athletes (NCAA 2020): division I athletes comprised 87% of the collegiate MLB draft picks, 85% of NBA rosters, 98% of NFL draft picks, and all NHL players with collegiate backgrounds. These disparities may serve as indirect evidence for the historically accepted differences in skill, competition and level of play in higher divisions.

In our analysis of over 100,000 hand and wrist injuries in collegiate athletes between 2004 and 2015, we found that the hand and wrist injury rates were significantly higher for division I student athletes compared to the other divisions; this was also consistent in both genders. Differences in certain performance variables and physical characteristics could serve as an explanation for our findings. Buell et al., evaluated body composition and characteristics of football linemen across NCAA divisions in 2008 and found that division I linemen (vs. division III) were 50lbs heavier and 4 inches taller. They also invested 3.5 h and 2.7 h more (per week) in resistance and cardiovascular training, respectively (Buell et al. 2008). Garstecki et al. compared Division I and division II football players and revealed that division I players were significantly stronger (bench press, squat, power clean), faster (40yd sprint) and leaner (body fat percentage) (Garstecki et al. 2004). Division I and division III football players were also compared and yielded similar results (Fry and Kraemer 1991). It is possible that the larger, faster and stronger division I athlete will generate more force with contact that translates to more injuries.

The discrepancy in available resources between divisions is substantial and may explain other findings central to our analysis. The size and scope of collegiate training facilities were evaluated in 2017. While the number of student athletes between division I and III was similar, division I institutions provided more central and satellite training facilities, each measuring greater than 2.5 × and 2 × the size, respectively (Gallucci and Petersen 2017). The largest division I programs spent 5 × the amount in dollars on a student athlete compared to division III programs (Rankin 1992). Greater available budgets can also translate to larger sports medicine staffs such as athletic trainers and strength and conditioning coaches. ATs at division III programs cared for almost three times more students than those at division I programs (Baugh et al. 2020). Athletic trainers and strength and conditioning coaches were associated with the implementation of injury prevention programs, and compliance is integral to the success these programs have in decreasing injuries (Silvers-Granelli et al. 2018; Hagglund et al. 2013; Joy et al. 2013). We revealed that division I athletes had the greatest number of NTL injuries and missed the fewest days from competition. Having less ATs available for student athletes could translate to poorer implementation and compliance to injury prevention programs. The majority of treatments provided by athletic trainers to injured athletes are for NTL rather than time loss injuries (Powell and Dompier 2004). It follows that a higher patient load for sports medicine staff could result in failing to identify potential NTL and subsequently prevent them from becoming time loss injuries (Powell and Dompier 2004). It is possible that if programs with less financial flexibility invest in more AT and supporting staff, they can increase the implementation and compliance to injury prevention programs and concurrently diagnose lesser injuries and prevent them from translating to more significant injuries. Staffing constraints has been cited as a major limitation for the implementation of concussion-related care policies (Baugh et al. 2015; Buckley et al. 2015).

The NCAA student athlete spends an average of 30–40 h per week, or the same amount of time as a full-time job, participating in their sport (NCAA. Goals study 2020). When an injury occurs, this may be devastating to team dynamics and to the student athlete. Narratives of victory on the playing field, respect for toughness and sacrifice, and the redemptive value of athletic participation are motivating factors for athletes to return to play following an injury (Corman et al. 2019). The degree of motivation varies by the student athlete and is reinforced by the tremendous amount of internal and external pressures for certain student athletes to compete in their sport. In the immediate setting, scholarships may be a major factor to return to play after injury. Scholarships are largely one-year agreements between the school and athlete (Athlete 2020). The decision to renew is re-evaluated yearly and is subject to withdrawal for reasons such as injuries and poor athletic performance. While division III athletes are not able to receive athletic scholarships, 50% of all division I athletes are receiving some degree of athletic scholarship. As such, the pressure on these athletes to stay healthy, perform at high levels and return to play are substantial.

The pressure to return to play is also driven by their future athletic ambitions. In 2019, collegiate athletes were polled on their perception of becoming a professional or Olympic athlete in their respective sport (NCAA. Goals study 2020). 76%, 76%, and 70% of division I ice hockey, basketball and football athletes, respectively, believed it was at-least somewhat likely to become a professional or Olympian. Alternatively, only 28% of male division III basketball players believed the same. Considering the attention that division I athletics receive from professional sporting teams, any playing time lost as a result of injury may negatively impact an athlete’s opportunity of becoming a professional.

These pressures may be manifested by the rates of surgery and time-lost following hand and wrist injuries. Division I athletes missed the fewest days overall, had the highest rates of NTL injuries yet the highest rate of surgery. While most hand and wrist injuries can be managed non-operatively (Wahl and Richard 2020; Geissler 2009), general indications for operative intervention exist such as displaced fractures, degree of angulation or malalignment. In certain settings, surgical intervention can be recommended for elite athletes with fractures that do not meet classical radiologic indications for surgery; it can offer faster recovery, decrease periods of joint immobilization and ultimately allow for faster return to play (Wahl and Richard 2020). It is possible that the internal pressure to play in elite college athletes could drive them to pursue surgery vs. athletes in other divisions with less incentive and pressures to return to the field. The options to pursue more aggressive management aimed at faster return to play should consider an athlete’s desires, demands and level of play (Wahl and Richard 2020; Halim and Weiss 2016). Also, in those divisions with more available trainers and physical therapists, athletes can receive the consistent rehabilitation that is required after pursuing such treatments (Geissler 2009).

There are several limitations of this study. Because we examined sports injuries collected in the NCAA-ISP database, our findings may not be generalizable to other sports or non-collegiate level athletics. We did not separate athletes based on which sport they played, nor by seniority, pre-, regular or post-season activity, player position or by playing surface. We did not look at specific injury mechanisms. When reporting the types of injuries sustained, we chose to present the general categories of injuries rather than the details of each fracture (e.g., 1st vs. 5th metacarpal fracture) or ligamentous injury (e.g., ulnar collateral ligament injury) as we felt this would provide a level of granularity that is beyond the scope of this manuscript. The data reported is from the NCAA-ISP database, which is based on voluntary reports from athletic trainers. This manner of data collection is vulnerable to variability between athletic trainers’ ability to identify injuries in their athletes, however, our large sample size would help mitigate this possible discrepancy. While this will continue to be a limitation for studies using this dataset, validation studies show that the NCAA-ISP captured 88.3% of all time-lost events across all divisions (Kerr et al. 2014; Kucera et al. 2011); this was then integrated into the weighting of the data provided by the ISP (Kerr et al. 2014). While using both eras of the dataset allowed us to increase our sample size, it should be noted that non-time-loss injuries were not recorded until the later era beginning in 2010. This change in data recording methodology affected all sports and divisions thus should not impact our general findings. Lastly, the analyses in this manuscript are based on weighted frequencies and thus the results have potential to be less precise if actual injury numbers are low. This will be a limitation for all research based on the ISP using its weighted frequencies.

In the future, we would like to evaluate hand and wrist injury rates in collegiate athletes over time to see if they are reduced, as the focus of the NCAA is on student athlete safety. It would also be interesting to look at surgical intervention rates over time in NCAA student athletes. Surgeons have become more aggressive about pursuing surgical management of hand and wrist injuries in some sports. In NCAA football in 2008, 6% of UCL injuries to the thumb underwent surgical intervention; in 2016, 10% were treated surgically (Carver et al. 2018). The focus should be on individualized treatment of collegiate athletes, emphasizing student athlete safety and preventing long-term complications.

## Conclusion

Hand and wrist injuries are common among collegiate athletes. Many of these injuries result in minimal time lost from participation, but severe injuries may require surgery and incur significant time away from athletics. NCAA division I athletes tend to be injured more than divisions II and III, with fewer missed days in male student athletes, and a higher overall surgical intervention rate. Further studies investigating the role of individual sports, player position, specific injury patterns and change in management over time are indicated.

## Data Availability

The NCAA Injury Surveillance Program data used in this study were provided by the Datalys Center for Sports Injury Research and Prevention.

## Abbreviations

AE: Athlete exposure
CI: Confidence interval
DRUJ: Distal radioulnar joint
ISP: Injury Surveillance Program
NCAA: National Collegiate Athletic Association
NCAA-ISP: National Collegiate Athletic Association Injury Surveillance Program
NTL: Non-time loss
TL: Time loss
TFCC: Triangular fibrocartilaginous complex
